# The Effect of Wind Exposure on the Web Characteristics of a Tetragnathid Orb Spider

**DOI:** 10.1007/s10905-017-9618-0

**Published:** 2017-05-18

**Authors:** Nicholas Tew, Thomas Hesselberg

**Affiliations:** 1 Department of Zoology, University of Oxford, South Parks Road, Oxford, OX1 3PS UK; 2 Department of Life Sciences, Imperial College London, Buckhurst Road, Ascot, SL5 7PY UK

**Keywords:** Wind damage, orb webs, behavioural plasticity, web geometry, forest edge effects

## Abstract

Studies on spiders in their natural habitats are necessary for determining the full range of plasticity in their web-building behaviour. Plasticity in web design is hypothesised to be important for spiders building in habitats where environmental conditions cause considerable web damage. Here we compared web characteristics of the orb spider *Metellina mengei* (Araneae, Tetragnathidae) in two different forest habitats differing in their wind exposure. We found a notable lack of differences in web geometry, orientation and inclination between webs built along an exposed forest edge and those built inside the forest, despite marked differences in wind speed. This suggests that *M. mengei* did not exhibit web-building plasticity in response to wind in the field, contrasting with the findings of laboratory studies on other species of orb spiders. Instead, differences in prey capture and wind damage trade-offs between habitats may provide an explanation for our results, indicating that different species employ different strategies to cope with environmental constraints.

## Introduction

Spiders face a crucial trade-off in their web-building behaviour between maximising prey capture and minimising energetic expenditure (Higgins 1995). Webs are intrinsically expensive to build, incurring costs associated with metabolism, silk production, time and predation risk (Peakall and Witt 1976; Prestwich 1977; Tanaka 1989; Pasquet et al. 1999). Web damage can impose a large fitness cost on spiders due to both reduced prey capture and the extra cost of repair or rebuilding behaviours (Chmiel et al. 2000; Wherry and Elwood 2009; Tew et al. 2015). Damage can result from a number of factors, including prey impact, larger non-prey animals, wind, rain and falling debris (Craig 1989; Chmiel et al. 2000; Walter and Elgar 2011). Wind, in particular, is known to be a major cause of web damage (Craig 1989), imposing relatively large aerodynamic drag forces which can cause radial and anchor threads to break and result in the partial collapse of the web (Lin et al. 1995; Zaera et al. 2014).

Orb spiders generally show a large degree of plasticity in their web design in response to habitat structure and size (Vollrath et al. 1997; Barrantes and Eberhard 2012; Hesselberg 2013), available prey (Schneider and Vollrath 1998; Venner et al. 2000; Blamires 2010) and environmental variables (Vollrath et al. 1997). Hence it is no surprise that several studies have found that spiders change their webs in response to wind. Laboratory studies on two species of the family Araneidae, *Araneus diadematus* (Hieber 1984; Vollrath et al. 1997) and *Cyclosa mulmeinensis* (Liao et al. 2009; Wu et al. 2013), have shown a reduction in web capture area when exposed to wind. A comparative field study of *Cyclosa ginnaga* and *C. mulmeinensis* revealed that much smaller webs were built by *C. mulmeinensis*, which inhabited windier environments (Liao et al. 2009). Reducing the area of the capture spiral appears to be an adaptation to windy conditions, presenting a lower surface area to the wind and producing a web that is more resistant to aerodynamic drag (Zaera et al. 2014).

The spacing, material properties and tension of silk threads are also influenced by wind. Orb webs built after exposure to artificial wind contained threads with larger spacing, higher tension and higher tensile strength (Vollrath et al. 1997; Liao et al. 2009; Wu et al. 2013). In the comparative field study of *Cyclosa* spp*.*, the species living in windier conditions, *C. mulmeinensis*, built webs of thicker, stronger and fewer radial threads, which also differed in terms of their amino acid composition (Liao et al. 2009). Webs with greater spacing in the capture spiral will sag less, with sticky threads being less likely to adhere to each other in windy conditions (Liao et al. 2009; Zaera et al. 2014).

Similarly, we might expect that the angle an orb web faces will have a bearing on wind-induced damages. There is some evidence that orb spiders orient webs parallel to the wind direction in both the laboratory (Hieber 1984) and the field (Schoener and Toft 1983; Ramirez et al. 2003). Building webs parallel to the wind, facing it side-on, allows spiders to minimise the surface area of the web that is exposed, and so reduces the potential for web damage. It has also been proposed that the inclination of an orb web, relative to the horizontal, is a feature that might be modified in response to wind (Eberhard 1971). A more horizontal web will face the wind at an angle that is closer to being side-on, and thus will have a lower surface area exposed. Studies on orb spiders that naturally construct vertical webs have not found evidence for this (Hieber 1984; Ramirez et al. 2003), but in orb webs with a more intermediate level of inclination, it could be a flexible feature.

Here we conducted a field study on the orb spider *Metellina mengei* (Blackwall 1869, family Tetragnathidae) to see whether webs were constructed differently between sheltered and exposed habitats. We chose to focus on *M. mengei* since it is a common species in European woodland and because it, like other tetragnathids, shows large natural variability in the inclination of its webs (N. Tew, Pers. Obs.). In addition, while there are numerous studies on webs from the family Araneidae, especially the genera *Araneus*, *Argiope* and *Cyclosa* (Zschokke and Herberstein 2005; Foelix 2011), studies on the other major orb spider family, the Tetragnathidae, are much rarer, and those on webs of the genus *Metellina* are absent from the published literature. We predicted that webs in more exposed habitats might have a smaller capture area, an orientation parallel to the prevailing wind and a more horizontal inclination. Finally, we investigated potential trade-offs between wind damage and prey capture in both habitats, given that prey capture in orb webs is known to be higher along an exposed forest edge (Vollrath 1985).

## Materials and Methods

### Study Species

*Metellina mengei* (previously known as *Meta mengei*) is a medium-sized orb-weaving spider (females: 3.5–6 mm, males: 3.5–5 mm) (Roberts 1996). It is widespread and abundant throughout Britain and much of Europe, with a broad habitat distribution and an annual life cycle. Females produce inclined orb webs and sit at the centre on the lower side, facing downwards (Roberts 1996). Spiders were visually identified in the field, but to confirm identification, 24 spiders were randomly collected during the study, stored in 70% ethanol, and later identified under a microscope. All collected specimens were identified as *M. mengei*.

## Study Site

Wytham Woods is a Site of Special Scientific Interest in Oxfordshire, UK, and is owned by the University of Oxford. All fieldwork was conducted in the Great Wood (51° 78′ N, 01° 34′ W), a 150 ha area of mixed deciduous woodland, which lies about six km north west of Oxford.

We selected two separate areas in the Great Wood, termed the sheltered and the exposed habitats. In both habitats, we marked three 200-m transects, each of which ran directly to one side of an infrequently-trodden path. The sheltered habitat was in the north east of the wood and transects in this area were well away from the edge (range 50 to 300 m). In contrast, the exposed habitat was along the western edge of the forest and the three transects here were only about five metres inside the wood (range 3 to 8 m). The understory was broadly similar in both habitats, although the vegetation tended to be shorter and less dense in the exposed habitat (average height exposed: ~42 cm vs. sheltered ~49 cm).

As expected, average wind speed during our study was significantly higher in the exposed (1.30 ± 0.13 ms^−1^, *N* = 60) than the sheltered habitat (0.03 ± 0.13 ms^−1^, *N* = 60; Welch t-test: *t* = 9.50, df = 60.6, *P* < 0.001), while neither temperature (14.5 ± 0.3 °C vs. 14.8 ± 0.2 °C, *N* = 60; Welch t-test: *t* = 0.88, df = 104.7, *P* = 0.38), pressure (1006 ± 1 vs. 1008 ± 1 mbar, *N* = 60; Welch t-test: *t* = 1.01, df = 115.8, *P* = 0.31) nor humidity (70.1 ± 1.9%RH vs. 67.5 ± 1.4%RH, *N* = 60; Welch t-test: *t* = 1.35, df = 105.7, *P* = 0.18) differed between the two habitats. Out of 60 measurements, 53 readings of zero were recorded for average wind speed in the sheltered habitat, compared with just six readings of zero in the sheltered habitat. The maximum wind speed recorded was 2.8 ms^−1^ in the sheltered habitat and 6.0 ms^−1^ in the exposed habitat. Wind direction did not differ significantly from a uniform distribution in the sheltered habitat (*n* = 10, $\overset{-}{\mathrm{R}}$=0.296, *p* = 0.428), but did in the exposed habitat (*n* = 54, $\overset{-}{\mathrm{R}}$=0.879, *p* < 0.0001), in which there was a clear westerly prevailing wind, with a circular mean of 263° W.

## Field Measurements

The study was carried out on ten days between 20-May-2015 and 02-Jun-2015. On each study day, we collected environmental and web data from both habitats, and all six transects. All three transects in one habitat were completed together before those in the other, but the order and direction in which habitats and transects were sampled varied systematically from day to day. The measurements were taken during the day, typically between 10.00 and 17.30, and all measurements were made well after sunrise (~05.00) and well before sunset (~21.00).

A set of environmental measurements was made twelve times per day, immediately before and after each of the six transects. Each set of measurements comprised the wind direction, average wind speed, maximum wind speed, temperature, atmospheric pressure and humidity, as well as the time. Wind direction was measured using a rudimentary wind sock and a compass. The wind was assumed to be predominantly horizontal as it had blown across flat fields before reaching the forest edge, an assumption that was supported by our wind sock. Average and maximum wind speed were measured over a two-minute period, with the anemometer held parallel to the horizontal wind direction about 0.5 m above ground (similar to the average web height). Shade temperature and air humidity were measured with an ETI Hygro-Thermo hygrometer and wind speed and barometric pressure with a Silva ADC Summit device.

All webs of adult and sub-adult *M. mengei* spiders along each transect, within 2 m to one side of the path, were measured, up until a limit of 20 webs per transect was reached. Webs of juvenile *Metellina* spiders were very rarely seen and easily recognised and ignored. Webs were sprayed lightly with water from a plant mister to allow them to be more visible when measured. To minimise any bias in locating webs, we walked back and forth along the paths, looking for webs from different heights and different search angles.

**Fig. 1:**
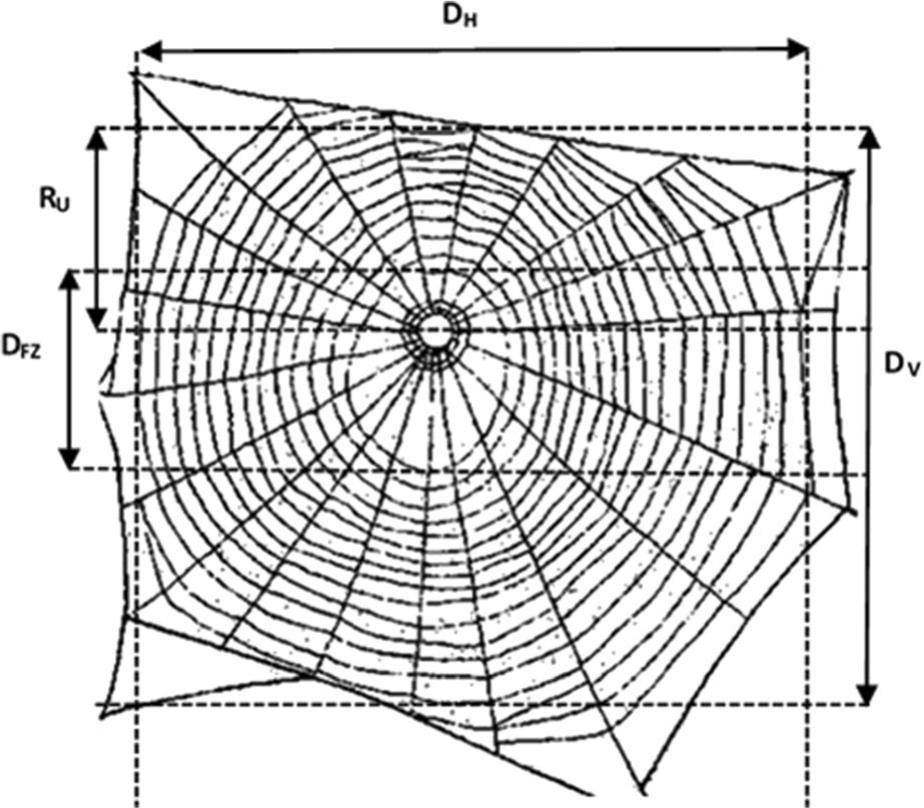
Diagram of a generic *Metellina* web, showing the upper radius (R_U_), the vertical (D_V_) and horizontal diameter (D_H_) as well as the diameter of the free zone (D_FZ_). The parameters were measured through the centre of the web, though they are shown at the edge in this schematic. The underlying *Metellina* web is reprinted by permission of HarperCollins Publishers Ltd. © Roberts 1996

We recorded five different web dimensions, four of which were measured directly to the nearest millimetre using a digital calliper (Fig. 1). The vertical (D_V_) and horizontal diameters (D_H_) of the capture spiral were measured between the outermost spiral threads, through the centre of the web. The upper radius of the capture spiral (R_U_) was measured from the hub straight to the uppermost spiral thread. The vertical diameter of the central hub and free zone area (D_FZ_) was also measured and the lower radius (R_L_) was calculated by subtracting the upper radius from the vertical diameter. From these values, we calculated vertical web asymmetry, with the formula: (R_U_ - R_L_) / (R_U_ + R_L_), and the area of the capture spiral (web area), with the Ellipse-Hub equation (Herberstein and Tso 2000):

$$ \mathrm{Web}\ \mathrm{area}=\uppi \left(\frac{{\mathrm{D}}_{\mathrm{V}}}{2}\right)\left(\frac{{\mathrm{D}}_{\mathrm{H}}}{2}\right)-\uppi {\left(\frac{{\mathrm{D}}_{\mathrm{FZ}}}{2}\right)}^2 $$

A further three web characteristics were recorded. Height was the vertical distance of the web hub from the ground, measured to the nearest centimetre using a tape measure. Inclination was measured to the nearest five degrees as an angle relative to the horizontal, using a protractor. Thus a completely horizontal web would be measured as 0° and a completely vertical web as 90°. Orientation was measured following Tolbert (1979) and quantified in degrees using a compass. Simply put, web orientation was defined as the horizontal direction in which the upper side of an inclined web faced. Finally, we counted the number of prey items in the web (prey capture). This value included prey stuck to the web and prey items in the web that the spider was currently eating.

## Data Analysis

The difference between the response variables web area, height, inclination and asymmetry in the sheltered and exposed habitats was analysed with liner mixed effect models (LMMs), including habitat as a fixed variable and study day and transect as random variables, using the *lmer* function from the R package ‘lme4’ (Bates et al. 2013). Logarithmic transformations were applied to web area, height and inclination in order to meet model assumptions. Prey capture was investigated with a generalised linear mixed model with a Poisson error distribution (GLMM-p) and including habitat, height, asymmetry, inclination, web area, temperature, humidity, pressure and selected interactions as fixed variables, as well as study day and transect as random variables, using the *glmer* function from the R package ‘lme4’. Non-significant terms were removed following the marginal rule to produce a final model with the lowest AIC score that included only significant terms (Thomas et al. 2013). The final model was found to have a weak overdispersion (θ = 1.4), which is within acceptable limits (Thomas et al. 2013).

Significance of the models was determined with Type II Wald Chi Square tests, as implemented in the function *Anova* from the R Package ‘car’ (Fox and Weisberg 2011), and models were validated against the assumptions of normality, heteroscedasticity, collinearity and autocorrelation (Thomas et al. 2013). The marginal coefficient of determination (R_m_^2^, summarising variance explained by fixed factors) and the conditional coefficient of determination (R_c_^2^, summarising variance explained by both fixed and random factors) were estimated for all significant models following the method described by Nakagawa and Schielzeth (2013), using the R package ‘arm’ (Gelman and Su 2014).

Directional data were analysed with a Rayleigh test from the ‘circular’ R package (Agostinelli and Lund 2013), with web orientation and wind direction data compared against a uniformly distributed null expectation. All statistical tests were carried out with R version 3.0.2 (R Core Team 2013), with the critical significance level set at 0.05. True independence was not present across the ten day study due to resampling of the same transects each day. Webs are often rebuilt overnight (Foelix 2011), in which case new webs would have been measured from one day to the next. However, it is still likely that individual spiders’ webs were surveyed multiple times during the duration of the study. The large sample size, combined with the use of mixed effects models, which considered study day and transect as random factors, means that the lack of true independence is unlikely to have compromised the reliability of the data analysis.

## Results

### Web Characteristics

**Fig. 2:**
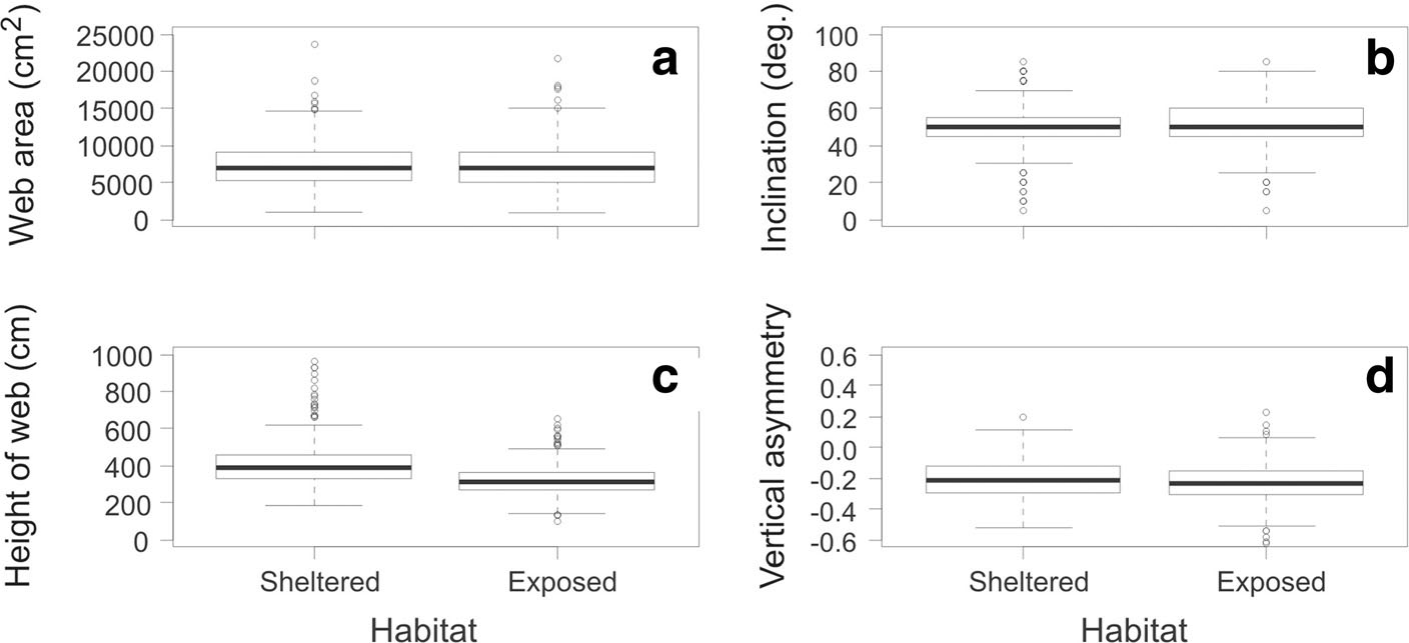
Characteristics of *Metellina mengei* webs in sheltered (sample size *n* = 279) and exposed habitats (sample size *n* = 430) of a forest. **a** Web area. **b** Web inclination. **c** Height of web above ground. **d** Vertical asymmetry of the web. The *bold horizontal line* indicates the median. The box encloses the first and third quartiles, while the whiskers extend to the most extreme data point within a distance of 1.5 times the interquartile range from the box. Data points outside this distance are indicated by *circles*

Across the ten-day study, 709 webs were measured, 279 in the sheltered habitat and 430 in the exposed habitat. Web area, inclination and asymmetry appeared very similar in the two habitats (Fig. 2a, b and d) and were not significantly different (LMM: Area: χ^2^ = 0.14, df = 1, *P* = 0.71; Inclination: χ^2^ = 1.85, df = 1, *P* = 0.17; Asymmetry: χ^2^ = 1.33, df = 1, *P* = 0.25). However, webs were built significantly higher above the ground in the sheltered compared to the exposed habitat (Fig. 2c, LMM: χ^2^ = 73.84, df = 1, *P* < 0.0001), with the model including random factors explaining 26% of the variation (R_c_^2^ = 0.257) and the habitat alone explaining 20% of the variation (R_m_^2^ = 0.204).

**Fig. 3:**
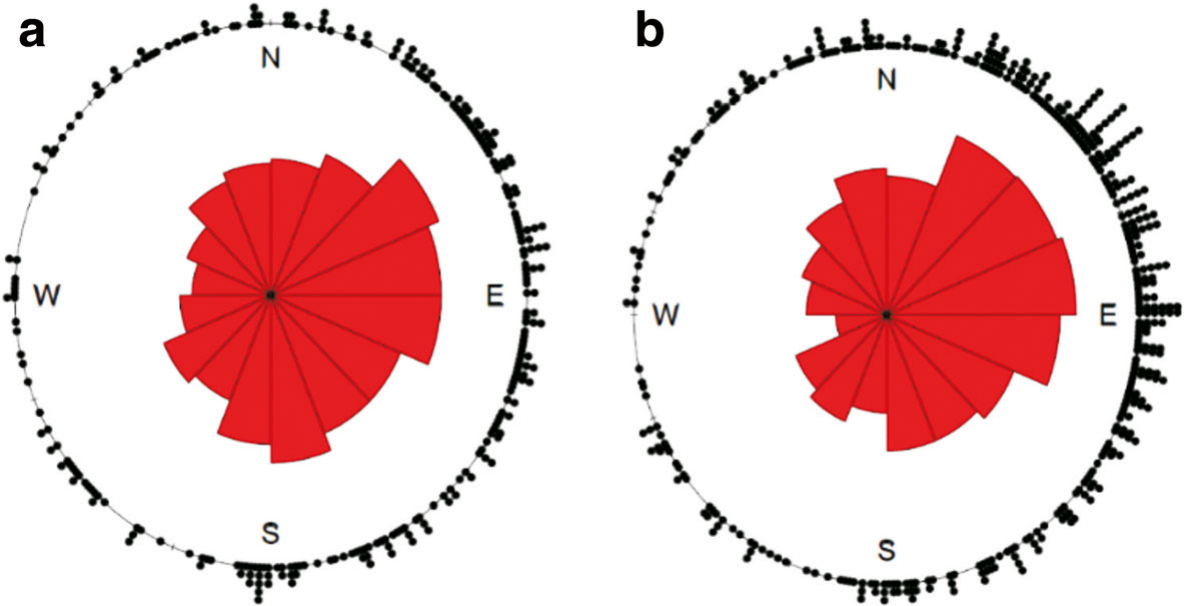
Circular histograms of *Metellina mengei* web orientation in the (**a**) sheltered (sample size *n* = 279) and (**b**) exposed habitats (*b*) (sample size *n* = 430) of a forest

The orientation of webs in both habitats differed significantly from a uniform distribution (Sheltered: *n* = 279, $\overset{-}{\mathrm{R}}$=0.275, *p* < 0.0001; Exposed: *n* = 430, $\overset{-}{\mathrm{R}}$=0.371, *p* < 0.0001), with more webs built in the eastwards 0° N to 180° S range and especially in the 45° NE to 135° SE range than would be expected if orientation were random (Fig. 3). The circular mean orientation in the sheltered habitat was 95° compared to 70° in the exposed habitat, but there was high variation around both of these values (Fig. 3). On average, in the exposed habitat, the orientation of webs per transect deviated from facing the wind by 193°, whilst no such value could be calculated for the sheltered habitat, in which transects experienced no prevailing wind. On average, the orientation of webs per transect deviated from facing the direction of the path by 48° in the sheltered habitat and 12° in the exposed habitat.

## Prey Capture

**Fig. 4:**
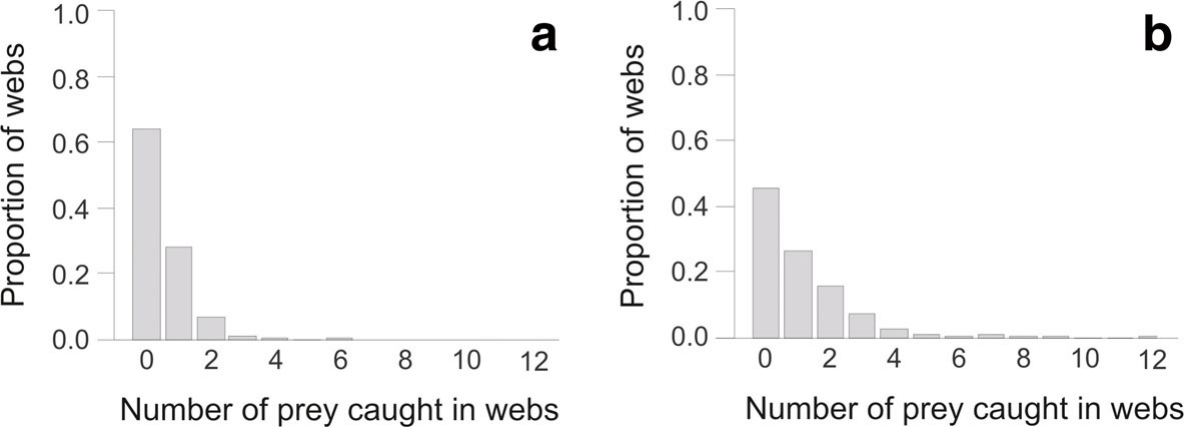
The distributions of prey capture in webs of *Metellina mengei* in the (**a**) sheltered (133 prey caught in 279 webs) and (**b**) exposed habitats (477 prey caught in 430 webs) of a forest

Significantly more prey were captured in the exposed than the sheltered habitat (GLMM-p: χ^2^ = 9.02, df = 1, *P* = 0.003). The mean number of prey captured in webs in the exposed habitat (1.11, SEM ±0.07) was more than double that in the sheltered habitat (0.48, SEM ±0.05). The distribution of prey capture in the exposed habitat was effectively shifted right compared to that in the sheltered habitat (Fig. 4), with a lower proportion of webs containing zero prey in the exposed habitat (0.45 vs. 0.64), and also a much greater proportion of them containing two or more prey items (0.29 vs. 0.08). In addition to habitat, web inclination (GLMM-p: χ^2^ = 8.15, df = 1, *P* = 0.004), web area (GLMM-p: χ^2^ = 16.43, df = 1, *P* < 0.0001) and mean daily temperature (GLMM-p: χ^2^ = 9.23, df = 1, *P* = 0.002) were found to be significant predictors of prey capture, all correlating positively. None of the other variables or interactions tested were significant (statistics not shown). The final model explained 30% of the variation (R_c_^2^ = 0.303), with the fixed effects alone explaining 20% of the variation (R_m_^2^ = 0.198).

## Discussion

A number of different environmental variables have been shown to influence features of orb webs in the laboratory, including wind, humidity and temperature (Vollrath et al. 1997; Liao et al. 2009). However, the adaptation of web characteristics to environmental variables in the field is poorly studied. Here we compared parameters of webs of the tetragnathid *Metellina mengei* between an inner sheltered and an outer exposed woodland habitat that differed only in wind exposure. Surprisingly, we found very little effect of wind on the webs.

In previous laboratory studies, wind speeds of 1.1 to 1.2 ms^−1^ were sufficient to cause significant changes to orb web structure and orientation (Hieber 1984; Liao et al. 2009; Wu et al. 2013), and in one study, a wind speed of only 0.5 ms^−1^ was sufficient (Vollrath et al. 1997). In this study we measured an average wind speed of 1.3 ms^−1^ in the exposed habitat, suggesting a similar treatment to that of webs in laboratory studies. The main difference is that we measured strong gusts of up to 6 ms^−1^ in the exposed habitat, in contrast to the laboratory studies, where wind speed was relatively constant. Such gusts are likely to cause web damage, considering that in one field study, a variable artificial airstream, with a velocity between 0.8 and 1.9 ms^−1^, resulted in considerable damage in just 20 s (Craig 1989). In addition, on very windy days, webs in the exposed habitat shook greatly and sometimes became damaged (N. Tew, Pers. Obs.). The combination of consistent moderate wind speeds and very strong gusts in the exposed habitat clearly imposed a pressure on webs. This pressure was not present in the sheltered habitat, which usually experienced a negligible wind speed and rarely any gusts.

## Web Characteristics

No significant differences were found in the geometry or angle of webs built in the two habitats. There was also no evidence that webs in the exposed habitat were more constrained in their structure, as similar variances in the measured variables were found in both habitats (Fig. 2). Thus, in our study, *M. mengei* females did not modify the size or shape of their webs in response to building in windier habitats. This result contrasts with published findings that smaller webs are built in more exposed habitats (Hieber 1984; Vollrath et al. 1997; Liao et al. 2009; Wu et al. 2013; Zaera et al. 2014). However, our results support the notion that different orb spider species can differ greatly in their web-building plasticity in response to environmental variables (Hesselberg 2015). Orb spiders are, for example, known to display markedly different abilities to adapt their webs to spatial constraints (Hesselberg 2013, 2014). This difference is even observed within a genus, as in one study, whilst *A. diadematus* halved its capture area in response to a 1.2 ms^−1^ airstream, *Araneus gemmoides* showed no such reduction (Hieber 1984). Instead of reducing its capture area, *A. gemmoides* oriented its web parallel to the wind flow and Hieber (1984) hypothesised that these contrasting strategies are alternative solutions to the same problem, and differ due to the precise species-specific trade-offs faced by the spiders in their natural habitats.

Webs, in our study, did not exhibit random orientation in either habitat, as the majority were facing eastwards in the 0° N to 180° S direction and especially in the 45° NE to 135° SE direction (Fig. 3). In the sheltered habitat, wind was rare, and when occasional gusts were measured, they had no prevailing direction. On the other hand, wind in the exposed habitat was strong and had a consistent westerly direction. On average, in this habitat, webs were built approximately perpendicular to the wind direction (deviating by 193°), not parallel to it (a deviation of 90°), as had been predicted from other studies (Schoener and Toft 1983; Hieber 1984; Ramirez et al. 2003).

In addition to minimising wind-induced damage, maximising prey capture is likely to be an important determinant of web orientation (Biere and Uetz 1981; Higgins and McGuinness 1991). One interpretation of the result that webs in the exposed habitat tended to face away from the prevailing wind is that this is an adaptation to increase prey capture, due to the non-random flight paths of insects (Cardé and Willis 2008). It is possible that, by presenting a large surface area of the web to the wind, more insects become stuck, either through upwind flight or being carried downwind. However, webs in both habitats tended to face towards the path, a similar result to that found by Tolbert (1979) in the araneid *Argiope trifasciata*. Again, this may be an adaptation to increase prey capture, as webs which face towards a more open area are likely to realise higher capture rates. This interpretation could explain why webs in the sheltered habitat exhibited non-random orientation (Fig. 3), despite the lack of a prevailing wind. Disentangling these two explanations is not possible from the existing data, but it seems that orb spiders may be able to use web-building plasticity to preferentially build in a direction that will bring about higher prey capture. This would be consistent with their ability to modify a host of other web characteristics in response to previous foraging performance (Venner et al. 2000; Blamires et al. 2011; Scharf et al. 2011).

There was no tendency for webs in the exposed habitat to be inclined nearer to the horizontal, in a way that would reduce the surface area exposed to wind, as predicted by Eberhard (1971). The webs in both habitats had very similar mean inclination values, at about 50° to the horizontal. There clearly was a capacity for webs to be angled much more horizontal or vertical than average, as webs were found to be inclined in the range 5° to 85° (with 95% of webs in the range 30°-75°). Web inclination is expected to affect foraging, with more vertically inclined webs obtaining higher prey capture in terrestrial habitats (Waldorf 1976; Opell et al. 2006), and horizontal webs built to catch prey alighting from low vegetation or emerging from water (Kato et al. 2003; Foelix 2011). This was also supported by our terrestrial study, as web inclination was a significant predictor of prey capture, correlating positively. On days when very strong gusts of wind were present in the exposed habitat, the shaking of vegetation was occasionally seen to damage webs (N. Tew, Pers. Obs). This raises the possibility that movement of the vegetation to which a web is anchored may be as important in effecting web damage during strong gusts as the orientation and inclination of the web to the wind.

The extent of vertical web asymmetry also did not differ between exposed and sheltered habitats in this study. Building asymmetric webs, with larger lower parts, is thought to be an adaptation to optimise prey capture in vertical orb webs, since spiders can run down faster than up due to gravity (Ap Rhiziart and Vollrath 1994; Heiling and Herberstein 1999; Nakata and Zschokke 2010), although a few spiders have reversed asymmetric webs (Eberhard 1975; Nakata and Zschokke 2010). In intermediately inclined webs, such as those in this study, asymmetry is likely to play less of a role. Height was the only web characteristic found to differ significantly between the two habitats, with webs in the exposed habitat being closer to the ground. It is possible that this is an adaptation to reduce wind damage as wind speeds are lower nearer to the ground, but it is most likely explained by the fact that the understory vegetation in the sheltered habitat tended to be taller (~49 cm) than in the exposed habitat (~42 cm).

## Prey Capture and Trade-Offs

Ultimately, the function of an orb spider’s web is to catch prey, and so prey capture is a key consideration in investigating any fitness trade-offs. Prey capture per web was significantly greater in the exposed habitat on the forest edge than the sheltered habitat inside the forest. This result is consistent with a previous study on *Nephila clavipes* (family Nephilidae), which found that individuals inside the forest grew at a slower rate than those at the forest edge (Vollrath 1985). In our study, prey capture in the exposed habitat was slightly greater with respect to the proportion of webs containing at least one prey item, and much greater considering webs with at least two (Fig. 4). It seems likely that flying insect abundance and activity was higher on the forest edge, leading to this higher prey capture (Jokimäki et al. 1998), although the link between higher prey activity and higher prey capture may not be strong, as a laboratory study found that spiders in windy conditions generally have a lower prey capture efficiency (Turner et al. 2011). In addition, many more webs were found in the exposed than in the sheltered habitat, again a result consistent with that of Vollrath (1985). The 20 web cap was reached for 18 of 30 transect surveys in the exposed habitat and only three in the sheltered one, suggesting that the forest edge, with its greater potential for prey capture, is a more popular habitat.

We postulate that there are three possible hypotheses for the lack of differences in web geometry, orientation and inclination between the two habitats: (1) It is possible that female *M. mengei* spiders lack the web-building plasticity required to construct their webs differently in a windier habitat. This seems unlikely as considerable behavioural flexibility has been shown in a number of different orb spider species and families (e.g. Vollrath et al. 1997; Blamires et al. 2011; Barrantes and Eberhard 2012; Wu et al. 2013), although this is not a universal trait (Hesselberg 2013, 2014).

(2) A second possibility is that the fitness trade-offs faced by the spiders differed between the two habitats. Spiders in the exposed woodland edge habitat paid greater costs associated with wind damage and possibly predation risk, as studies on insects indicate that predation and parasitism risk can be higher at the forest edge compared to the forest interior (McGeoch and Gaston 2000; Rossetti et al. 2013). However, these spiders might be able to afford such costs due to obtaining higher prey capture rates (Vollrath 1985). The geometry, orientation and inclination of the web may therefore maximise prey capture, rather than minimise web damage, explaining the lack of a reduction in capture area, the intermediate inclination and the orientation towards the wind and the path.

Daily rebuilding and relocation are two other important considerations. Many diurnal orb spiders take down their web each night, often ingesting the silk to conserve energy (Breed et al. 1964; Carico 1986). The decision whether to rebuild and/or relocate the web probably depends upon prey capture and web damage obtained during the preceding day (Gillespie 1987; Chmiel et al. 2000). Thus in the exposed habitat, spiders may spend more time in the vegetation without having a web, compared to the sheltered habitat, thus reducing the costs of web building and predation risk. The maximum of 20 webs was found for each transect in the exposed habitat for the first six days of the study. The final four days had poorer weather, characterised by lower temperatures, rain and higher wind speeds. During these days the number of webs was reduced dramatically to an average of around six per transect. Perhaps these spiders could afford to ‘sit out’ periods of poor weather because of their high level of recent prey capture, and only rebuild their webs when they need to feed again.

(3) Finally, it is possible that web modifications were made, but in features not investigated in this study. For example, the number, spacing, strength and tension of silk threads have all been shown to be modified in response to wind (Vollrath et al. 1997; Liao et al. 2009; Wu et al. 2013), but these features were not investigated in this field study. The number and spacing of radial and spiral threads did not appear to differ strongly between the two habitats (N. Tew, Pers. Obs.), but no estimations could be made about differences in their strength and tension.

More data on prey availability, web damage and silk material properties are needed, but hypothesis 2 provides the most likely explanation of our results. The lack of clear adaptations to the higher wind regime in the exposed habitat is most likely due to trade-offs between web damage and prey capture rates.

## Conclusion

This study investigated web-building plasticity in response to wind in a variety of web features in natural habitats in an understudied tetragnathid spider. A very limited degree of web-building plasticity in response to wind was observed in *M. mengei*. Instead of wind and associated web damage, prey capture efficiency may be a more important consideration, with respect to modifications in web geometry, orientation and inclination.

The lack of clear behavioural plasticity reported in this study is surprising in the light of previous laboratory studies (Hieber 1984; Vollrath et al. 1997; Wu et al. 2013) and highlights the importance of combining laboratory studies with field studies. However, our results support the hypothesis that different species of orb spider show different degrees of plasticity in their web-building behaviour and employ different strategies to similar environmental factors (Hesselberg 2014, 2015).

## References

[CR1] Agostinelli C, Lund U (2013) R package 'circular': circular statistics (version 0.4-7). https://r-forge.r-project.org/projects/circular/. Accessed 01 February 2017

[CR2] Ap Rhiziart A, Vollrath F (1994). Design features of the orb web of the spider, *Araneus diadematus*. Behav Ecol.

[CR3] Barrantes G, Eberhard WG (2012). Extreme behavioral adjustments by an orb-web spider to restricted spaces. Ethology.

[CR4] Bates D, Maechler M, Bolker B, Walker S (2013) lme4: linear mixed-effects models using Eigen and S4. R package version 1.0-5. http://CRAN.R-project.org/package=lme4. Accessed 01 February 2017

[CR5] Biere JM, Uetz GW (1981). Web orientation in the spider *Micrathena gracilis* (Araneae: Araneidae). Ecology.

[CR6] Blamires SJ (2010). Plasticity in extended phenotypes: orb web architectural responses to variations in prey parameters. J Exp Biol.

[CR7] Blamires SJ, Chao Y-C, Liao C-P, Tso I-M (2011). Multiple prey cues induce foraging flexibility in a trap-building predator. Anim Behav.

[CR8] Breed AL, Levine VD, Peakall DB, Witt PN (1964). The fate of the intact orb web of the spider *Araneus diadematus*. Behaviour.

[CR9] Cardé RT, Willis MA (2008). Navigational strategies used by insects to find distant, wind-borne sources of odor. J Chem Ecol.

[CR10] Carico JE, Shear WA (1986). Web removal patterns in orb-weaving spiders. Spiders: web behaviour and evolution (pp 306–318).

[CR11] Chmiel K, Herberstein M, Elgar M (2000). Web damage and feeding experience influence web site tenacity in the orb-web spider *Argiope keyserlingi* Karsch. Anim Behav.

[CR12] Craig CL (1989). Alternative foraging modes of orb web weaving spiders. Biotropica.

[CR13] Eberhard WG (1971) The ecology of the web of *Uloborus diversus* (Araneae: Uloboridae) 10.1007/BF00389107 28310979

[CR14] Eberhard WG (1975). The ‘inverted ladder’ orb web of *Scoloderus* sp. and the intermediate orb of *Eustala* (?) sp. Araneae: Araneidae. J Nat Hist.

[CR15] Foelix RF (2011). Biology of spiders.

[CR16] Fox J, Weisberg S (2011). An R companion to applied regression.

[CR17] Gelman A, Su Y-S (2014) Arm: data analysis using regression and multilevel/hierarchical models. R package version 1.7-07. http://CRAN.R-project.org/package=arm. Accessed 01 February 2017

[CR18] Gillespie RG (1987). The mechanism of habitat selection in the long-jawed orb-weaving spider *Tetragnatha elongata* (Araneae, Tetragnathidae). J Arachnol.

[CR19] Heiling AM, Herberstein ME (1999). The role of experience in web-building spiders (Araneidae). Anim Cogn.

[CR20] Herberstein ME, Tso I-M (2000). Evaluation of formulae to estimate the capture area and mesh height of orb webs (Araneoidea, Araneae). J Arachnol.

[CR21] Hesselberg T (2013). Web-building flexibility differs in two spatially constrained orb spiders. J Insect Behav.

[CR22] Hesselberg T (2014). The mechanism behind plasticity of web-building behavior in an orb spider facing spatial constraints. J Arachnol.

[CR23] Hesselberg T (2015). Exploration behaviour and behavioural flexibility in orb-web spiders: a review. Curr Zool.

[CR24] Hieber CS (1984). Orb-web orientation and modification by the spiders *Araneus diadematus* and *Araneus gemmoides* (Araneae: Araneidae) in response to wind and light. Z Tierpsychol.

[CR25] Higgins LE (1995). Direct evidence for trade-offs between foraging and growth in a juvenile spider. J Arachnol.

[CR26] Higgins L, McGuinness K (1991). Web orientation by *Nephila clavipes* in Southeastern Texas. Am Midl Nat.

[CR27] Jokimäki J, Huhta E, Itämies J, Rahko P (1998). Distribution of arthropods in relation to forest patch size, edge, and stand characteristics. Can J For Res.

[CR28] Kato C, Iwata T, Nakano S, Kishi D (2003). Dynamics of aquatic insect flux affects distribution of riparian web-building spiders. Oikos.

[CR29] Liao C-P, Chi K-J, Tso I-M (2009). The effects of wind on trap structural and material properties of a sit-and-wait predator. Behav Ecol.

[CR30] Lin LH, Edmonds DT, Vollrath F (1995). Structural engineering of an orb-spider’s web. Nature.

[CR31] McGeoch MA, Gaston KJ (2000). Edge effects on the prevalence and mortality factors of *Phytomyza ilicis* (Diptera, Agromyzidae) in a suburban woodland. Ecol Lett.

[CR32] Nakagawa S, Schielzeth H (2013) A general and simple method for obtaining *R*^*2*^ from generalized linear mixed-effects models. Methods Ecol Evol 4:133–142

[CR33] Nakata K, Zschokke S (2010). Upside-down spiders build upside-down orb webs: web asymmetry, spider orientation and running speeds in *Cyclosa*. Proc R Soc Lond [Biol].

[CR34] Opell BD, Bond JE, Warner DA (2006). The effects of capture spiral composition and orb-web orientation on prey interception. Zoology.

[CR35] Pasquet A, Leborgne R, Lubin Y (1999). Previous foraging success influences web building in the spider *Stegodyphus lineatus* (Eresidae). Behav Ecol.

[CR36] Peakall DB, Witt PN (1976). The energy budget of an orb web-building spider. Comp Biochem Phys A.

[CR37] Prestwich KN (1977). The energetics of web-building in spiders. Comp Biochem Phys A.

[CR38] R Core Team (2013). R: a language and environment for statistical computing.

[CR39] Ramirez MG, Wall EA, Medina M (2003). Web orientation of the banded garden spider *Argiope trifasciata* (Araneae, Araneidae) in a Californian coastal population. J Arachnol.

[CR40] Roberts MJ (1996). Collins field guide to spiders of Britain and Northern Europe.

[CR41] Rossetti MR, Salvo A, Videla M, Valladares G (2013). Forest remnants contribute to parasitoid conservation: experimental evaluation of parasitism on a leafminer host. J Insect Conserv.

[CR42] Scharf I, Lubin Y, Ovadia O (2011). Foraging decisions and behavioural flexibility in trap-building predators: a review. Biol Rev Camb Philos Soc.

[CR43] Schneider JM, Vollrath F (1998). The effect of prey type on the geometry of the capture web of *Araneus diadematus*. Naturwissenschaften.

[CR44] Schoener TW, Toft CA (1983). Dispersion of a small-island population of the spider *Metepeira datona* (Araneae: Araneidae) in relation to web-site availability. Behav Ecol Sociobiol.

[CR45] Tanaka K (1989). Energetic cost of web construction and its effect on web relocation in the web-building spider *Agelena limbata*. Oecologia.

[CR46] Tew ER, Adamson A, Hesselberg T (2015). The web repair behaviour of an orb spider. Anim Behav.

[CR47] Thomas R, Vaughan I, Lello J (2013) Data analysis with R statistical software. A guidebook for scientists. Eco-explore

[CR48] Tolbert WW (1979). Thermal stress of the orb-weaving spider *Argiope trifasciata* (Araneae). Oikos.

[CR49] Turner J, Vollrath F, Hesselberg T (2011). Wind speed affects prey-catching behaviour in an orb web spider. Naturwissenschaften.

[CR50] Venner S, Pasquet A, Leborgne R (2000). Web-building behavior in the orb-weaving spider *Zygiella x-notata*: influence of experience. Anim Behav.

[CR51] Vollrath F (1985). Web spider’s dilemma: a risky move or site dependent growth. Oecologia.

[CR52] Vollrath F, Downes M, Krackow S (1997). Design variability in web geometry of an orb-weaving spider. Physiol Behav.

[CR53] Waldorf ES (1976). Spider size, microhabitat selection, and use of food. Am Midl Nat.

[CR54] Walter A, Elgar MA (2011). Signals for damage control: web decorations *in Argiope keyserlingi* (Araneae: Araneidae). Behav Ecol Sociobiol.

[CR55] Wherry T, Elwood RW (2009). Relocation, reproduction and remaining alive in an orb-web spider. J Zool.

[CR56] Wu C-C, Blamires SJ, Wu C-L, Tso I-M (2013). Wind induces variations in spider web geometry and sticky spiral droplet volume. J Exp Biol.

[CR57] Zaera R, Soler A, Teus J (2014). Uncovering changes in spider orb-web topology owing to aerodynamic effects. J R Soc Interface.

[CR58] Zschokke S, Herberstein ME (2005). Laboratory methods for maintaining and studying web-building spiders. J Arachnol.

